# Bacterial Diversity in Oral Samples of Children in Niger with Acute Noma, Acute Necrotizing Gingivitis, and Healthy Controls

**DOI:** 10.1371/journal.pntd.0001556

**Published:** 2012-03-06

**Authors:** Ignacio Bolivar, Katrine Whiteson, Benoît Stadelmann, Denise Baratti-Mayer, Yann Gizard, Andrea Mombelli, Didier Pittet, Jacques Schrenzel, The Geneva Study Group on Noma (GESNOMA)

**Affiliations:** 1 Institut für Angewandte Immunologie, Zuchwil, Switzerland; 2 GESNOMA, Unit of Plastic and Reconstructive Surgery, University of Geneva Hospitals, Geneva, Switzerland; 3 Genomic Research Laboratory, University of Geneva Hospitals, Geneva, Switzerland; 4 Department of Periodontology and Oral Pathophysiology, School of Dental Medicine, University of Geneva Faculty of Medicine, Geneva, Switzerland; 5 Clinical Microbiology Laboratory, University of Geneva Hospitals, Geneva, Switzerland; 6 Infection Control Program and World Health Organization Collaborating Centre on Patient Safety, University of Geneva Hospitals and Faculty of Medicine, Geneva, Switzerland; University of Tennessee, United States of America

## Abstract

**Background:**

Noma is a gangrenous disease that leads to severe disfigurement of the face with high morbidity and mortality, but its etiology remains unknown. Young children in developing countries are almost exclusively affected. The purpose of the study was to record and compare bacterial diversity in oral samples from children with or without acute noma or acute necrotizing gingivitis from a defined geographical region in Niger by culture-independent molecular methods.

**Methods and Principal Findings:**

Gingival samples from 23 healthy children, nine children with acute necrotizing gingivitis, and 23 children with acute noma (both healthy and diseased oral sites) were amplified using “universal” PCR primers for the 16 S rRNA gene and pooled according to category (noma, healthy, or acute necrotizing gingivitis), gender, and site status (diseased or control site). Seven libraries were generated. A total of 1237 partial 16 S rRNA sequences representing 339 bacterial species or phylotypes at a 98–99% identity level were obtained. Analysis of bacterial composition and frequency showed that diseased (noma or acute necrotizing gingivitis) and healthy site bacterial communities are composed of similar bacteria, but differ in the prevalence of a limited group of phylotypes. Large increases in counts of *Prevotella intermedia* and members of the *Peptostreptococcus* genus are associated with disease. In contrast, no clear-cut differences were found between noma and non-noma libraries.

**Conclusions:**

Similarities between acute necrotizing gingivitis and noma samples support the hypothesis that the disease could evolve from acute necrotizing gingivitis in certain children for reasons still to be elucidated. This study revealed oral microbiological patterns associated with noma and acute necrotizing gingivitis, but no evidence was found for a specific infection-triggering agent.

## Author Summary

Noma is a devastating gangrenous disease that leads to severe facial disfigurement, but its cause remains unknown. It is associated with high morbidity and mortality and affects almost exclusively young children living in remote areas of developing countries, particularly in Africa. Several factors have been linked to the disease, including malnutrition, immune dysfunction, lack of oral hygiene, and lesions of the mucosal gingival barrier, particularly the presence of acute necrotizing gingivitis, and a potentially non-identified bacterial factor acting as a trigger for the disease. This study assessed the total bacterial diversity present in 69 oral samples of 55 children in Niger with or without acute noma or acute necrotizing gingivitis using culture-independent molecular methods. Analysis of bacterial composition and frequency showed that diseased and healthy site bacterial communities are composed of similar bacteria, but differ in the prevalence of a limited group of phylotypes. We failed to identify a causative infectious agent for noma or acute necrotizing gingivitis as the most plausible pathogens for both conditions were present also in sizeable numbers in healthy subjects. Most likely, the disease is initiated by a synergistic combination of several bacterial species, and not a single agent.

## Introduction

Noma (cancrum oris) is a rapid and disfiguring gangrenous disease of the face with high morbidity and mortality [1]. Although it affects young children in developing countries almost exclusively, it was widespread before access to vaccines and improved nutrition became common. Noma has also been observed in HIV-infected individuals and patients with leukemia in developed countries, and occurred in concentration camps during World War II. There are no documented cases of other children living in the same village, siblings, or even identical twins developing noma at the same time, and it is impossible to transfer the infection to animal models [1]. Several factors have been linked to the disease, including malnutrition, immune dysfunction, lack of oral hygiene, and lesions of the mucosal gingival barrier, particularly the presence of acute necrotizing gingivitis (ANG) [2]–[4]. However, the etiology of noma remains poorly understood [1]–[3], [5]–[8].

While it seems reasonable to assume that noma is a multifactorial disease, it has long been suggested that bacteria are essential to the development of this condition. Noma infections respond to antibiotic treatment and the type of necrosis and odor of the lesions have been identified as bacterial in origin [1]. Studies have reported the presence of certain types of microorganisms in samples taken from noma cases. These include *Prevotella melaninogenica*, *Actinomyces pyogenes*, *Fusobacterium nucleatum*, *Bacteroides fragilis*, *Bacillus cereus*, *P. intermedia*, and *F. necrophorum* [5], [6], [9], with the latter two considered by some authors as significant pathogens in the etiology of noma. A study of advanced noma lesions in four children in Nigeria sequenced 212 16 s rRNA genes using culture-independent methods and found a great diversity of known oral and novel microbes, including those normally associated with soil and other non-human environments [10]. However, no clear association of a particular species with disease was observed in these patients.

Often identified in all forms of periodontal disease and necrotizing gingivitis, *P. intermedia* has been viewed as a potential causative pathogen due to its frequent recovery from noma lesions using conventional culture techniques [5], [6]. *F. necrophorum*, a predominant animal pathogen, has attracted particular attention because of its association with necrotizing infections in both humans and animals. Anaerobic bacteria including *Fusobacterium* spp. and *Prevotella* spp. are known to become established in human infant mouths at around the same time as tooth eruption [11]. In at least one study, *F. necrophorum* was not found in the normal microbiota in malnourished Nigerian children without noma [9], while it was identified by culture methods in affected children [5], [9]. It is found in the gut of herbivores and can persist in wet soil with a high manure content on land used by cattle and sheep [12]. *F. necrophorum* infections develop in livestock when skin is damaged by wet or cold conditions, during tooth eruption, and other circumstances [12]–[14]. Fecal contamination in populations living close to cattle could be a source of *F. necrophorum* in noma infections [6].

Establishing the role of specific microorganisms in the pathogenesis of noma is currently limited by three problems. First, it is likely that the use of culture techniques has led to an underestimation of the diversity of the bacterial microbiota given the difficulty with growing a large spectrum of fastidious organisms. Second, the fact that the disease develops rapidly and is predominantly observed in remote areas where access to medical care and facilities is difficult precluded the study of early cases. Microorganisms found in great quantity in advanced lesions may represent changes after the disease develops, rather than its cause. Third, since this disease occurs in populations where even the normal oral microbiota is poorly investigated, it remains unclear if an organism is related to the disease or simply belongs to the normal microbiota of the investigated cohort and reflects a particular lifestyle, socioeconomic status, or geographic location.

To advance our knowledge of the etiology of noma, it is essential to obtain access to early acute cases, to study the microbiota of related conditions in parallel, e.g., ANG and healthy controls, and to apply advanced microbiological techniques to study the diversity of the sampled microbiota. The Geneva Study Group on Noma (GESNOMA) is an extensive network of medical professionals, biologists, and community-based collaborators composed of two teams, one located in Geneva, Switzerland, and the other in Zinder, Niger. The Geneva team [15] includes specialists in the fields of general dentistry, periodontology, epidemiology, pediatrics, infectiology, virology, biology, genomics, and plastic and reconstructive surgery. The Niger team comprises two nurses trained in the diagnosis of noma and sampling techniques, and a driver.

The aim of the microbiological part of the GESNOMA project, which began in September 2001, is to elucidate the role of microorganisms in noma. Here, we present the results of our first attempt to inventory and compare the bacterial diversity in oral samples of children with acute noma and ANG with healthy controls using culture-independent phylogenetic techniques [15]. To the best of our knowledge, this study is the first to investigate acute noma cases and compare them with age-matched controls originating from the same environment.

## Methods

### Subjects

Children were seen in a consulting center for noma care in the region of Zinder, Niger, created by the non-governmental humanitarian organization “Sentinelles”. Following an extensive information campaign conducted among the local population, a considerable number of acute cases were admitted to the center for treatment. We included 23 children less than 12 years old with acute noma. For each acute noma case, four control children without noma matched for age were recruited from the same village. For the present analysis, we selected the best age-matched control of the same sex. In addition, we also included nine children with ANG who were referred to the center. Every child underwent general, facial, and oral examination to establish the clinical diagnosis with particular attention paid to the presence of spontaneous bleeding, gingival ulceration, pseudo-membranes, halitosis, and gingival pain. Informed consent was obtained from parents/guardian and the study was approved by the Ministry of Health, Republic of Niger.

ANG diagnosis was defined by the presence of the following three pathognomonic signs/symptoms: pain, spontaneous bleeding, and gingival ulceration (decapitation of gingival papilla). Acute noma was diagnosed when the underlying bone was visible intra-orally. Extra-orally, the children could present facial edema or necrosis. Children with old lesions, antibiotic treatment, hypernutrition, or having dental care during the previous three months were excluded.

Each child diagnosed with acute noma received treatment at the noma center in Zinder. The median delay between the first signs of noma and our examination was 11 days. Treatment consisted of restoring general health (rehydration, reintroduction of proper nutrition, vitamin supplementation, antibiotics, and transfusion if needed), together with wound care and early physiotherapy to avoid mouth stricture, the typical sequelae of noma characterized by contracting fibrous scars that often interfere with mouth movement, eating, and speech [1]. When the acute phase was over, the child left the center and went back to his/her village where the “Sentinelles” social workers shared information and advice about noma to facilitate their reintegration to normal life. Each child consulting for noma at the Zinder center is followed up for 10 years (some undergo operations in Niger during surgical missions, others in Europe, and a few will not require surgery).

### Microbiological sampling

Microbial samples were taken from the subgingival plaque of teeth in the premolar/deciduous molar region. One sample was taken from each of the controls (n = 23) and ANG subjects (n = 9). In the noma cases, subgingival plaque samples were obtained from one diseased site (n = 23) and one site in another dentition area without clinical signs of disease (n = 23). When it was impossible to obtain a subgingival sample from a tooth in the region of the noma lesion, a sample was taken from the marginal region of the wound instead. The study teeth were dried and isolated with cotton rolls to avoid salivary contamination. Two sterile paper points were sequentially inserted into the same gingival crevice. Samples were immediately suspended in guanidinium thiocyanate extraction buffer and frozen at −20°C.

### Sample preparation and amplification

The 78 samples obtained were prepared for polymerase chain reaction (PCR) as follows: tubes were heated for 10 min at 60°C and the paper point was removed. Isopropanol was added to 50% and the samples were precipitated for 2 h at −20°C. After centrifugation, precipitates were washed in 70% ethanol and dried and redissolved in 100 µl of 0.5 M NaOH for 10 min at 70°C. One µl of the NaOH solution was diluted in 100 µl of 100 mM Tris buffer pH 8 [16].

For PCR, 1 µl of the Tris solution was amplified in a 25 µl reaction containing 1 unit of Taq DNA polymerase (Roche Diagnostics, Basel, Switzerland), 2.5 µl of 10× Mg^++^ reaction buffer (Roche Diagnostics), the 4 dNTPs at a final concentration of 200 mM each, and amplification primers at a final concentration of 0.4 mM each. Amplification consisted of 35 cycles of denaturation for 15 sec at 94°C, annealing for 20 sec at 50°C, and elongation for 2 min at 72°C, followed by a final 10 min elongation at 72°C. Amplification primers were broad-specificity fD1 and rP1 [17] as described, but used here without the polylinker sequences (fD1: AGAGTTTGATCCTGGGTCAG, rP1: ACGGTTACCTTGTTACGACTT). The forward primer matches 172,490 of the 1.5 million bacterial rRNA gene sequences in version 10 of the Ribosomal Database Project, while the reverse primer matches 21,204 from this database [18]. All reactions were aliquoted from one single master mix and performed simultaneously on the same cycler to ensure PCR conditions as uniform as possible. PCR results were inspected by agarose gel electrophoresis and 69 reactions that showed a clean, one-band amplification product of the required size were retained for library construction.

### Library construction

Seven libraries were prepared as summarized in Table 1. For all samples to be equally represented in the libraries, the individual reactions were diluted according to the visual intensity of the amplified fragment to approximately the same DNA concentration. Aliquots of the reactions were then mixed in seven pools, precipitated, redissolved, and purified by excision of the fD1-rP1 fragment on a preparative agarose electrophoresis gel. While pooling the samples will eliminate individual variation from the results and may allow one unusual sample to bias these, it was necessary to make the project manageable considering the resources and manpower of a single laboratory before the widespread availability of high-throughput sequencing. A comparison of bacterial species composition in the male and female sample groups, which was not expected to be different, provided a meaningful control. Fragments were cloned in the pGEM®-T vector (Promega Corporation, Madison, WI, USA). Two hundred plasmid minipreps per library were one-shot sequenced with the fD1 primer on an ABI 3100 capillary system and 1285 sequences of about 500 bp were obtained in the 5′ end of the molecule. Chimeric sequences were identified using Blast [19] home-made scripts and with manual inspection of suspect sequence alignments. The full fD1-rP1 fragment of about 1500 bp was additionally sequenced for a subset of 339 selected sequences. Sequences of all phylotypes reported in this study were deposited in the GenBank nucleotide sequence database under accession numbers AM419953 through AM4202291 All analyses were performed with the Statistica package (STATISTICA data analysis software system, version 7.1. 2005, StatSoft Inc., Tulsa, OK, USA).

**Table 1:** Library description.

Child status	Sex	Site status	Library number	No. of children	Mean age	Validsequences
Noma	Female	Diseased	1	10	2.9	180
		Healthy	2	9	3	168
	Male	Diseased	3	13	4.7	192
		Healthy	4	11	4.7	182
Healthy	Female	Healthy	5	8	3.5	181
	Male	Healthy	6	10	5.4	165
ANG	Both	Diseased	7	8	5.1	169

### Phylogenetic analyses

Phylogenetic analyses were conducted on the full 16 S rRNA dataset containing 339 sequences each about 1500 bases long. Probabilistic methods were used to reconstruct phylogenetic trees with Maximum Likelihood as implemented by PHYML version 2.4.4 [20], [21] and Bayesian inferences as implemented by MrBayes 3.1.2 [20]. Hierarchical likelihood ratio tests performed with Modeltest 3.04 [22]–[24] indicated that a General Time Reversible model with rate variation among sites and a proportion of invariable sites (GTR+Γ8+I) was the best-fit model of nucleotide substitution for the dataset.

In the PHYML procedure, the starting tree was obtained with BIONJ [25], which is an improved version of the neighbor-joining algorithm of Saitou and Nei [26]. The parameters of the GTR+Γ8+I model were estimated and optimized during the search. Bayesian posterior probabilities were calculated using a Metropolis-coupled, Markov chain, Monte Carlo sampling approach as implemented by MrBAYES 3.1.2 and using the same GTR+Γ8+I model.

Two runs of four simultaneous Markov chains each were performed for two million generations with trees sampled every 10 generations. After approximately 20,000 generations, the log-likelihoods of trees reached an asymptote. These initial trees were discarded as burn-in. Posterior probabilities (pp) were subsequently computed from the consensus of the remaining 180,000 sampled trees. MrBAYES was set to estimate model parameters during the full optimization.

Sequences that were 99% to 100% similar to a fully-named GenBank sequence were given the GenBank species name in the trees, except in some cases where the GenBank sequence was evidently misclassified. The remaining phylotypes were ascribed to higher level taxonomic divisions based on tree topology. The use of full-length 16 s rRNA gene sequences for each phylotype representative facilitated this process. A separate tree was built for Fusobacteria by combining GenBank representatives of this taxon with our fusobacterial phylotypes.

## Results

### Library description

Short, defective, or chimeric sequences were eliminated, reducing the dataset to 1242 sequences. All represented bacterial 16 S rRNAs, except for five Lactuca chloroplast sequences. The final dataset comprised 1237 sequences grouped in 339 phylotypes by blasting them against each other, including those in the Human Oral Microbe Database (HOMD) and GenBank (Table S1). A phylotype, as defined by Paster et al. [27], describes a sequence that differs from known species by approximately 30 bases (or 2%) and is at least 99% similar to other members of the cluster. Eighty-five percent of these phylotypes were >97% similar to sequences already present in the HOMD database. Full fD1-rP1 fragment 16 s rRNA gene sequences of 1400–1500 bp were obtained for most phylotype representatives.

The predictor of Boneh [28] applied to the species frequency data resulted in an estimate of 81 unseen species yielding a total bacterial diversity of 423 species in the population. By contrast, the ACE predictor of Chao and Lee [29], as implemented in the SPADE program [30], estimated the total diversity in our population at 600 species. The latter estimate may be more realistic because ACE gave accurate predictions of the actual diversity observed in our sample when applied to random subsets of the data (600 and 900 samplings), while the Boneh predictor grossly underestimated it.

Table 2 lists the bacterial taxa found. The 339 phylotypes were distributed in 8 phyla. By comparing the number of phylotypes in each family with the total number of occurrences, the heterogeneity of each family can be estimated. For instance, only 9 phylotypes of Peptostreptococcaceae accounted for 78 occurrences of this family, while the 69 occurrences of Porphyromonadaceae were distributed in 22 phylotypes, indicating a more than three-fold larger diversity. In addition, Table 2 shows the percentage of occurrences of each major family in the three libraries representing diseased sites.

**Table 2:** Taxonomic distribution of the phylotypes.

Phylum	Class	Order	Family	Number of phylotypes	Total occurrences	% in dis-eased sites*
Actinobacteria	Actinobacteria	Actinomycetales	Actinomycetaceae	3	4	
			Corynebacteriaceae	4	5	
			Micrococcaceae	1	1	
			Propionibacteriaceae	2	14	0%
	Other			1	6	
Bacteroidetes	Bacteroidetes	Bacteroidales	Porphyromonadaceae	22	69	43%
			Prevotellaceae	59	295	72%
			Other	1	1	
	Flavobacteria	Flavobacteriales	Flavobacteriaceae	18	45	5%
	Other			7	19	83%
TM7 phylum				2	3	
Deferribacteres			Deferribacteraceae	5	8	
Firmicutes	bacilli	Bacillales	Staphylococcaceae	5	33	22%
		Lactobacillales	Aerococcaceae	3	6	
			Carnobacteriaceae	3	5	
			Lactobacillaceae	1	1	
			Leuconostocaceae	1	5	
			Streptococcaceae	26	212	35%
	Clostridia	Clostridiales	Acidaminococcaceae	41	99	45%
			Eubacteriaceae	14	29	64%
			Lachnospiraceae	17	43	62%
			Peptococcaceae	1	2	
			Peptostreptococcaceae	6	52	86%
			Other	11	19	53%
	Mollicutes	Anaeroplasmatales	Erysipelotrichaceae	2	2	
		Mycoplasmatales	Mycoplasmataceae	1	1	
		Other		1	1	
Fusobacteria			Fusobacteriaceae	31	108	44%
Proteobacteria	Alphaproteobacteria	Rhodobacterales	Rhodobacteraceae	1	1	
		Other		2	2	
	Betaproteobacteria	Burkholderiales	Burkholderiaceae	6	23	16%
		Neisseriales	Neisseriaceae	11	56	28%
	Deltaproteobacteria	Desulfobacterales	Desulfobulbaceae	1	1	
	Epsilonproteobacteria	Campylobacterales	Campylobacteraceae	4	9	
	Gammaproteobacteria	Cardiobacteriales	Cardiobacteriaceae	1	1	
		Pasteurellales	Pasteurellaceae	9	33	22%
		Pseudomonadales	Moraxellaceae	2	2	
			Pseudomonadaceae	1	4	
		Other		1	2	
Spirochaetes			Spirochaetaceae	11	15	84%
Total				339	1237	

*The percent in diseased sites is only shown for families present at more than 1% of the total number of sequences * The percent in diseased sites is only shown for families present at more than 1% of the total number of sequences (1237).

### Statistical data analysis

**Figure 1:**
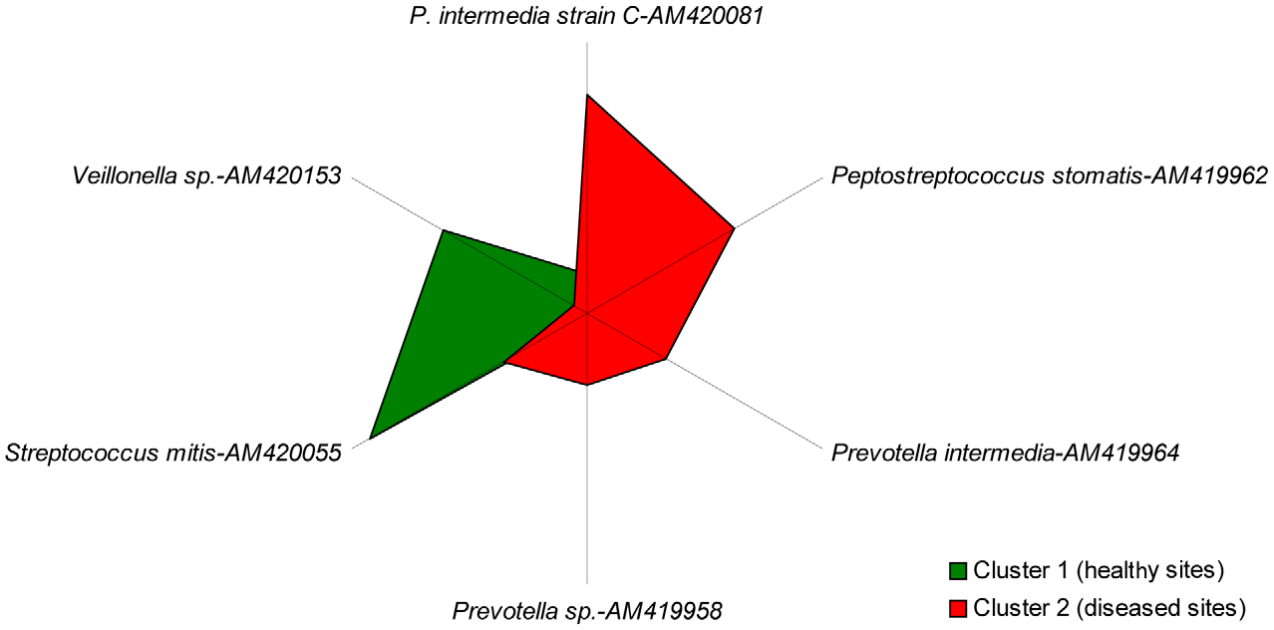
Radial plot of means for significant bacterial species or phylotypes in K-means clustering. Polygons of means for healthy and diseased sites are colored in green and red, respectively.

The bacterial microbiota of libraries was compared using statistical methods to search for significant patterns of organization. As a first step, the seven libraries were subjected to K-means cluster analysis. When the number of desired clusters was set to 2, a group was obtained that comprised libraries 2, 4, 5 and 6, which were all healthy site libraries, while the second group comprised the diseased site libraries 1, 3 and 7. In *post hoc* variance analysis, six bacterial species showed significant differences between group distribution at the 95% or more probability level (Table 3 and Figure 1). High frequencies for some *Prevotella* and *Peptostreptococcus* species were associated with the diseased status. When the desired number of clusters was set to 3 or more, the *post hoc* variance analysis gave no significant results.

**Table 3:** Statistics for significant bacteria in K-means clustering.

Clone	AccessionNo.	Species	F	Probability	Cluster means	
					1	2
101B10	AM419958	*Prevotella sp.*	7.14	0.044	0.25	3.33
101D05	AM419962	*Peptostreptococcus stomatis*	8.73	0.032	1.25	8.00
101E01	AM419964	*Prevotella intermedia*	8.17	0.035	0.25	4.00
202H12	AM420055	*Streptococcus pneumoniae*	9.44	0.028	11.25	4.33
302B03	AM420081	*Prevotella intermedia* strain C	7.06	0.045	1.75	10.33
402G08	AM420153	*Veillonella parvula* oral clone	6.71	0.049	7.50	0.66

**Figure 2:**
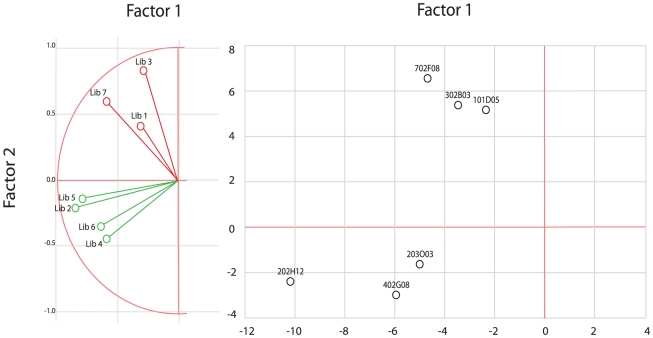
Plot of the libraries and the most discriminant bacterial species by principal component analysis. A: Libraries from healthy and diseased sites are colored green and red, respectively. B: Bacterial species or phylotypes are color-coded according to their weight in healthy (green) and diseased (red) libraries.

For principal component analysis (PPCA), the two first eigenvalues were retained, accounting for 56% of the variability in the data. The contribution of major bacteria to these eigenvalues is listed in Table 4. Two factors were computed: factor 1 had a large contribution from libraries 2, 4, 5, 6 and 7, characterized by the absence of a noma lesion, while factor 2 had a high contribution from libraries 1, 3, and 7, characterized by disease status, and either noma or ANG. Similar to cluster analysis, PPCA clearly separated the healthy and diseased conditions (Figure 2A). The structure of factor 1 suggests that the microbial microbiota of noma and ANG could be distinguished in principle. In Figure 2B, the six bacterial species or phylotypes contributing the most to each factor are plotted on the factor plane.

**Table 4:** Statistics for major bacteria in principal component analysis.

Clone	Accessionnumber	Species	Percent contributions to	
			Factor 1	Factor 2
202H12	AM420055	Streptococcus pneumoniae*	33.3	2.8
402G08	AM420153	Veillonella parvula oral clone*	11.4	4.2
203003	AM420056	*Prevotella genomosp.*	8.4	1.3
702F08	AM420266	*Prevotella intermedia strain 2*	7.2	20.4
302B03	AM420081	Prevotella intermedia strain C*	3.7	14.9
101D05	AM419962	Peptostreptococcus stomatis*	1.9	14.0
		Cumulative contribution to factors	65.9	57.6

Species labelled * are significant in k-means clustering.

Additional computations were performed with the aim to understand more precisely the difference between the healthy and diseased site libraries. The three diseased site libraries (1,3,7) harbor a total of 202 phylotypes out of 541 sequences; the four healthy site libraries (2,4,5,6) harbor 221 phylotypes out of 696 sequences and, hence, are of similar diversity. There are 84 shared phylotypes comprising all abundant phylotypes (>1% of total sequences), which account for 62% of all observations. The similarity between the two groups was quantified by computing two different indexes as described [31] and implemented [30] by Chao et al. The adjusted abundance similarity indexes of Jaccard and Sorensen were 0.76 and 0.87, respectively. Both computations indicated that the total shared species (observed plus unseen) constituted 100% of the healthy site microbiota and about 75% of the diseased site microbiota. These figures confirm the observation that healthy and diseased site libraries differ in quantitative, rather than qualitative terms.

The preceding analyses emphasize the somewhat expected differences between healthy and diseased site bacterial microbiota, but do not provide any clues for identifying putative differences between the noma and non-noma children. To eliminate the healthy/diseased site variability from the analyses, the male and female libraries were combined for all healthy and all diseased sites. Analyses were then performed on the resulting five bacterial sets: [1+2], [3+4], 5, 6 and 7.

K-means clustering grouped the libraries according to the noma ([1+2],[3+4]) and non-noma (5,6,7) dichotomy. In *post hoc* variance analysis, seven phylotypes showed between-group distribution differences significant at the 95% probability level; however, four have a very low prevalence and failed to pass the binomial distribution test at the same probability level. The most significant of the remaining three phylotypes was *P. intermedia*, AM420081, already one of the significant phylotypes in HS/DS analysis. In contrast to other *Prevotella*, this one is in low abundance in the ANG library and therefore forces the grouping of noma versus non-noma libraries. When this single phylotype was excluded from the analysis, K-means clustering failed to separate the noma from the non-noma libraries. The two remaining phylotypes with different representation in the healthy and diseased groups were *Neisseria sicca* and *Veillonella sp*. *N. mucosa* AM419960 ranks eighth in the list of the most prevalent species and constitutes 3.6% of the microbiota in noma children and less than 0.8% in non-noma children, with a similar representation in the healthy and disease site samples. *V. parvula* AM420153 ranks fourth in total prevalence and constitutes 3.2% of the noma children microbiota (mostly in healthy sites) and 1.7% of the control plus ANG children microbiota. Using PPCA, the analysis did not differ markedly from the results of the healthy/disease site analysis after merging the [1+2] and [3+4] gender groupings; the noma and ANG libraries were grouped apart from the healthy control libraries.

### Phylogenetic analyses

The MrBAYES tree summarizing the phylogenetic relationships of all 339 phylotypes is depicted in the supplementary material (Figure S1). The tree obtained with Phyml was very similar. The topology obtained was highly robust with more than 90% of the nodes supported with a probability of 95% or more. The current taxonomical classification of the bacteria (Phyla, classes, order, genus) is respected. We give the taxonomic identification of each phylotype to the highest level of identification possible throughout the text and in the tree in Figure S1 (i.e. when the identification is not possible past the family level, it is assumed that the sequences belong to the range of possible identities inside that family).

The phylotypes found in the statistical analysis to characterize the healthy and diseased conditions are highlighted in green and red, respectively. For all remaining phylotypes occurring five times or more, we computed the binomial distribution probability that the phylotype was not distributed randomly between the healthy and diseased site library sets. When the probability was significant (higher than 95%) the phylotype was colored green or red. A total of 40 phylotypes were color-coded. Of note, of the number of phylotypes tested at the 95% probability level, only five to six are expected to be colored by chance. We tried to disclose the higher level taxonomic divisions that were associated with clinical status by browsing the tree from outside in and computing for each highly supported node containing a colored phylotype the binomial distribution probability that the combined clade of phylotypes descending from the node was not equally distributed between the healthy and diseased site libraries. The node was colored green or red when this probability was significant and the node did not comprise lower level nodes or phylotypes of conflicting color.

**Figure 3:**
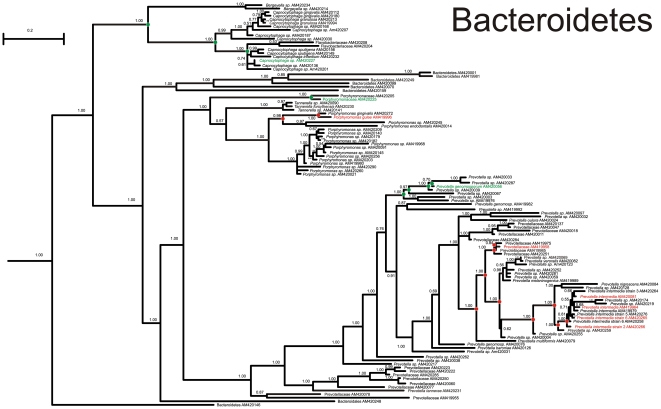
MrBAYES tree of the Prevotellaceae family. Nodes with a statistical support (posterior probability) > = 95% are circled. Phylotypes and highly supported nodes are colored green or red according to their prevalence in healthy or diseased sites, respectively (see text for details).

At the end of the procedure, several clades were highly correlated with the clinical condition of the sites. In the phylum Bacteroidetes, a clade belonging to the Prevotellaceae (Figure 3) comprising *P. intermedia*, *P. melaninogenica*, *P. verolalis*, *P. nigrescens*, and *P. multiformis* was associated with disease. The whole class Flavobacteria, the genus *Tannerella*, and another clade of Prevotellaceae were associated with healthy sites. Among the phylum Proteobacteria, the alpha, beta, gamma and delta-proteobacteria were associated with healthy sites. Among the Firmicutes, the genus *Peptostreptococcus* was strongly associated with diseased sites, and the genus *Staphylococcus* with healthy sites, as well as mixed behavior.

**Figure 4:**
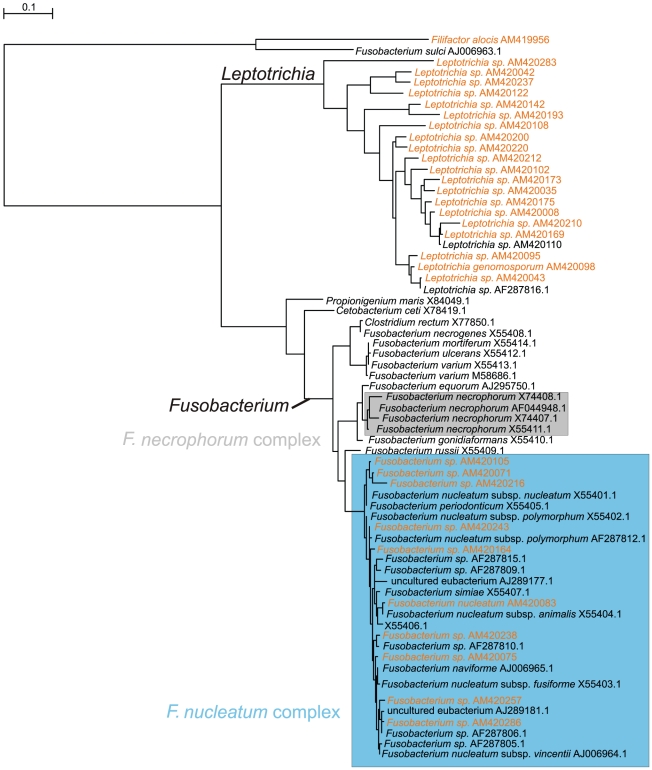
MrBAYES tree of the *Fusobacterium* genus. Phylotypes found in our study are colored red. The *F. necrophorum* complex is shown as a grey box.

As *F. necrophorum* has been suspected to be associated with, or a causative agent of noma, special attention was given to the Fusobacteria by constructing a separate tree (Figure 4) with our phylotypes and GenBank representatives of the genus *Fusobacterium*. It appeared that all 10 phylotypes of the genus found in our study belong to the *F. nucleatum* complex. The *Fusobacterium* genus as a whole constituted 6% of the bacterial microbiota, both in healthy and diseased sites. In the statistical analyses, phylotypes were distributed without healthy/diseased site or noma/non-noma bias.

## Discussion

As a first step to elucidate the role of microorganisms in the etiology of noma, we have assessed the total bacterial diversity in 69 oral samples from 55 children living in the same geographical region in Niger, Africa, with or without acute noma or ANG, using culture-independent molecular methods. A total of 339 bacterial species or phylotypes were identified from 1237 partial 16 s rRNA gene sequences. Culture techniques may lead to an underestimation of the diversity due to difficulties with growing fastidious organisms, and are potentially misleading in understanding the true etiology of a multifactorial condition. Our use of direct sequencing and the generation of full-length 16 S sequences shed new light on the overall differences between healthy and disease states of noma. While the number of sequences per sample is much less than that found in high throughput sequencing studies, 10 sequences per sample are enough to distinguish between sample groups in many cases [32]. For infections that are overwhelmed by a particular pathogen, this number of sequences would be enough to determine which bacteria dominated infected samples.

All samples were obtained using the paper point method, a commonly-used procedure for sampling the subgingival microbiota. Although other techniques, such as sampling with curettes, may be advantageous in certain situations (e.g., collection of firmly attached biofilm on tooth surfaces for the study of caries), paper points offered the possibility of uniform sampling in all target sites, including ulcers.

Our data obtained from healthy children and those with acute noma can be compared with those obtained by Paster et al [33] from adult North American subjects with different periodontal conditions using a similar methodology. Paster detected 347 phylotypes from 2522 clones. Approximately 40% were novel phylotypes. By comparing our phylotypes with the 302 phylotypes of the Paster collection available in the GenBank database, we found that 144 had a practically identical match. The shared phylotypes accounted for 61% of the total subgingival bacterial microbiota in the Niger children. Two- thirds or more of the subgingival bacterial microbiota of Niger children are composed of bacterial species also present in North American adults. We used also BLAST to compare our 339 sequences with 20 novel sequences obtained from four children with advanced noma lesions and published in 2002 by Paster et al (Table S2) [10]. Seventeen of the 20 sequences had alignments of >1300 bp with identities ranging from 73–87%, including a *Treponema* species unique to noma infections (clone BZ013), which had 83% identity to an uncultured *Treponema* species from our study (AM420013). Both studies have a small number of sequences per sample and different biases, including the fact that the wounds in the Paster study [10] were older (6 weeks to 2 years compared to 11 days in the current study). However, it is not surprising that both studies found increasing numbers of new and different species.

Statistical analysis showed that significant differences exist between the microbiota of healthy and diseased sites, but not between noma and non-noma sites. The finding that noma cannot be distinguished from ANG corroborates the prevailing view that noma evolves from, or is triggered by this condition. Bacterial microbiota characteristic of disease differs from healthy microbiota by a series of quantitative modifications, among which the most striking are strong increases in the counts of some *Prevotella* and *Peptostreptococcus* phylotypes. Further study to understand why these particular species were more prevalent in the disease state could be important. Even species with identical 16 S sequences have a very different potential for virulence.

Spirochetes as a group are also strongly associated with disease (Table 2). In studies using culture-based techniques, microscopy or primers specific for Spirochetes, a large diversity and high proportion of Spirochetes – as much as half – have been associated with periodontal disease [34]–[37]. Experimental bias either at the DNA extraction or the PCR amplification step may have led to underrepresentation of Spirochetes. Our PCR primers are capable of amplifying Spirochetes; the forward primer matches 602 out of the 6192 Spirochetes from the 1.6 million sequences in the Ribosomal Database Project, and the reverse primer matches 46 out of the 6192 Spirochetes. However, the reverse primer requires at least one error to match *T. denticola* and other examples of uncultivated oral *Treponema*.

ANG was first described as a fusiform-spirochete infection, but it was later recognized that *P. intermedia* is the predominant microorganism in the lesions [5], [6]. Working with ANG- and noma-afflicted Nigerian children, Falkler et al. [5], [6] found a predominance of *P. intermedia* in both conditions. Our study confirms and gives quantitative support to these observations and points to a possible role for *Peptostreptococcus* as well.

While we have a larger number of subjects with acute noma wounds, the average number of days that passed since the appearance of the noma wound before sampling was 11 days, meaning that there may be heterogeneity in the stage of the infection in these samples. Microbes that trigger the infection may be different from those that appear later in the disease process. The complexity introduced by a weakened immune system means that an opportunistic pathogen may be powerful in this context. Noma infections have the unusual ability to penetrate bone, although we and others have not found large numbers of novel bacteria that could explain this unusual destructive ability. The important role of a weakened immune system may explain how noma infections can penetrate bone and why it does not spread between individuals living in close contact. Future studies that include direct sampling of the bone surface at the site of infection, especially with metagenomic sequencing of all genes in the sample, may reveal which organism or enzymes are responsible for bone destruction.

Direct studies of microbial communities will all be biased toward the inclusion of particular species, usually depending on the method of DNA extraction and PCR amplification. All conclusions drawn from this study require a comparison with identically-treated samples. Systematic errors may universally exclude some species, but we are confident that the differences that arise between samples are subject to the same bias.

It must be emphasized that the technique of library cloning produces ratios of species instead of absolute numbers. An increase or a decrease in the representation of any species is matched automatically by a decrease/increase for some other species. In our case, it cannot be concluded from the statistical analysis that the increase in numbers of *Prevotella*, for instance, plays a role in disease — it could well be that the leading event is rather that the numbers of other taxa, such as *Capnocytophaga* or *Streptococcus,* have decreased. ANG and noma could result from an excess of injuring agents, a lack of protective agents, or from both. Our study shows only what is the “normal” healthy microbiota among local controls and quantifies changes in community composition in the disease state.

Apart from *P. intermedia*, Falkler et al. detected *F. necrophorum*, generally a livestock pathogen, in most noma patients [5], [6] in one study [9], but only in one of 30 healthy children in another study [9]. This finding led the authors to hypothesize that this microorganism could be responsible for the onset of noma. In our study, Fusobacteria were a major component of the subgingival bacterial microbiota in both healthy and diseased children without any difference, either quantitative or qualitative, between the two groups. Furthermore, all the phylotypes of the genus *Fusobacterium* that were identified belong to the *F. nucleatum* complex. Therefore, our data do not support the hypothesis that *F. necrophorum* plays a significant role in the etiology of noma.

To the best of our knowledge, this study is the first to investigate acute noma cases and compare them with age-matched controls originating from the same environment. We failed to identify a causative infectious agent for noma or ANG since the most plausible pathogens for both conditions were also present in sizeable numbers in the healthy subjects. Most likely, the disease is initiated not by a single agent, but by a synergistic combination of several bacterial species in the context of poor immune function. The relationship between organisms in a complex community is difficult to assess. Ecologists paired up with microbiologists have made promising attempts at assessing species interdependence in human oral samples using co-occurence analysis [38]. Clinical examples of treating polymicrobial infections, such as cystic fibrosis, by eliminating a member of the community that normally colonizes asymptomatically reveal that understanding microbial community dynamics is more complex than simply identifying the most common microbe in the infection [39]. With only 16 S data, and pooled samples, it would be difficult to identify or explain this kind of co-occurence in our data. One promising idea is to use comparative genomics from full genome sequence data from species that are found to be co-occurring to identify shared functions, which may explain the co-occurrence [40], [41]. Time-resolved studies with time points before and after infection, along with immunological assessment of noma-affected and control children, would both shed light on the origins of this condition.

## Supporting Information

Figure S1 **MrBAYES tree of identified phylotypes.Nodes with a statistical support (posterior probability) > = 95% are circled.** Phylotypes and highly supported nodes are colored green or red according to their prevalence in healthy or diseased sites, respectively (see text for details).
(EPS)

Table S1 **BLAST results against the Human Oral Microbe Database (HOMD) and Genbank.**
(XLS)

Table S2 **BLAST results showing similarity between 20 novel 16 S sequences from Paster et al 2002** [**10**] **and our 339 phylotypes.**
(DOC)
