# Supplementary material for: Bacterial Diversity in Oral Samples of Children in Niger with Acute Noma, Acute Necrotizing Gingivitis, and Healthy Controls
Source: PLoS Negl Trop Dis. 2012 Mar 6;6(3):e1556. doi: 10.1371/journal.pntd.0001556 (PMC3295795; doi:10.1371/journal.pntd.0001556)
Supplement: Table S2 — BLAST results showing similarity between 20 novel 16 S sequences from Paster et al 2002 [10] and our 339 phylotypes. (DOC) [file pntd.0001556.s003.doc]

**Supplemental Table 2. BLAST results showing similarity between 20 novel 16S sequences from Paster et al 2002 and our 339 phylotypes.**

| **Query ID from Paster 2002** | **Hit from 339 phylotypes reported here** | **Length** | **Identities** |
| --- | --- | --- | --- |
| **S000344653 Caryophanon sp. oral clone AW086; AF385535** | S000944699 uncultured Granulicatella sp.; 701G07(oral); AM420261 | 1684 | 80.5 |
| **S000344646 Leptothrix sp. oral clone AV011a; AF385528** | S000944478 uncultured Lautropia sp.; 202B04(oral); AM420040 | 1681 | 80.6 |
| **S000344652 Leptothrix sp. oral clone AW043; AF385534** | S000944478 uncultured Lautropia sp.; 202B04(oral); AM420040 | 1679 | 81.7 |
| **S000344660 Peptostreptococcus sp. oral clone FG014; AF385543** | S000944511 uncultured Peptostreptococcus sp.; 301F09(oral); AM420073 | 1678 | 86.7 |
| **S000345114 Porphyromonas sp. oral clone AW032; AF393476** | S000944647 uncultured Porphyromonas sp.; 601A05(oral); AM420209 | 1655 | 86.6 |
| **S000344655 Holophaga sp. oral clone CA002; AF385537** | S000944600 uncultured Veillonella sp.; 501B12(oral); AM420162 | 1552 | 73.7 |
| **S000344658 Paenibacillus sp. oral clone CA007; AF385540** | S000944531 uncultured Streptococcus sp.; 302F04(oral); AM420093 | 1548 | 78.7 |
| **S000344656 beta proteobacterium oral clone CA004; AF385538** | S000944478 uncultured Lautropia sp.; 202B04(oral); AM420040 | 1545 | 82.8 |
| **S000344654 Treponema sp. oral clone BZ013; AF385536** | S000944451 uncultured Treponema sp.; 101D06(oral); AM420013 | 1542 | 83.5 |
| **S000344650 Kocuria sp. oral clone AW006; AF385532** | S000944682 uncultured Rothia sp.; 603015(oral); AM420244 | 1539 | 84.8 |
| **S000344648 Caryophanon sp. oral clone AV085; AF385530** | S000944559 uncultured Staphylococcus sp.; 401E07(oral); AM420121 | 1402 | 80.2 |
| **S000344659 Leptotrichia sp. oral clone FB074; AF385542** | S000944560 uncultured Leptotrichia sp.; 401E08(oral); AM420122 | 1398 | 81.1 |
| **S000344651 Sphingomonas sp. oral clone AW030; AF385533** | S000944552 uncultured alpha proteobacterium; 303H06(oral); AM420114 | 1394 | 79.1 |
| **S000344647 Sphingomonas sp. oral clone AV069; AF385529** | S000944552 uncultured alpha proteobacterium; 303H06(oral); AM420114 | 1391 | 79.2 |
| **S000344645 Microbacterium sp. oral clone AV005b; AF385527** | S000944682 uncultured Rothia sp.; 603015(oral); AM420244 | 1390 | 81.9 |
| **S000344657 Bdellovibrio sp. oral clone CA006; AF385539** | S000944563 uncultured Burkholderiaceae bacterium; 401F06(oral); AM420125 | 1379 | 74.4 |
| **S000344649 Pedobacter sp. oral clone AV100; Av100; AF385531** | S000944672 uncultured Bergeyella sp.; 602D02(oral); AM420234 | 1314 | 74.9 |
| **S000345117 Paenibacillus sp. oral clone BZ008; AF393479** | S000944614 uncultured Abiotrophia sp.; 501F02(oral); AM420176 | 454 | 79.1 |
| **S000345115 alpha proteobacterium sp. oral clone AW149; AF393477** | S000944552 uncultured alpha proteobacterium; 303H06(oral); AM420114 | 388 | 84.0 |
